# Supplementary material for: Adherence to an Injury Prevention Warm-Up Program in Children’s Soccer—A Secondary Analysis of a Randomized Controlled Trial
Source: Int J Environ Res Public Health. 2021 Dec 13;18(24):13134. doi: 10.3390/ijerph182413134 (PMC8701740; doi:10.3390/ijerph182413134)
Supplement: Supplementary file 1 [file ijerph-18-13134-s001.zip › Figure S1.pdf]

Figure S1. NEUROMUSCULAR TRAINING WARM UP PROGRAM

Each warm-up session contains general warm-up (1), strength exercises for hip, trunk and lower extremities (2,3,5), a balance exercise (4) and speed & deceleration drills (6,7). There are different variations of each exercise with diverse difficulty and different need of space. As the players' abilities improve the coach can keep challenging them with more demanding exercises.

1. General Warm-up (Aerobic & Coordination)

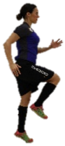

5–15 meters back and forth or 10–20 repetitions in place (if limited space), 2–4 sets.
- First, forward run & change of direction technique
- Second, choose one of the following exercises (a, b, c):
  a. Forward run with backward zig-zag
  b. Jumping Jacks forward-backward or sideways
  c. Skipping forward-backward or sideways

2. Hip Muscle Activation (Strength)

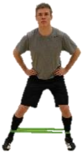

6–10 repetitions or 3–6 m back and forth, 2–4 sets.
Place a mini-band around both legs, right below the knee
or above the ankle.
- Choose one of the following exercises (a, b, c):
  a. Squat with heel lifts
  b. Squat jump
  c. Monster Walk forward-backward or sideways

3. Plank (Strength)

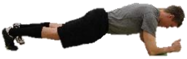

4–6 slow repetitions, 2–4 sets.
- Choose one of the following exercises (a, b, c):
  a. Front plank
  b. Side plank
  c. Push up

4. Single Leg Balance (Balance)

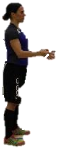

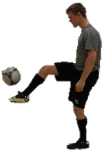

4–6 slow repetitions per leg, 2–4 sets.
With or without football
Choose one of the following exercises (a, b, c):
  a. Single leg hip-hinge
  b. Single leg balance with ball pass (kick)
  c. Single leg balance with ball pass (toss/roll)

5. Lunges or Split Squat (Strength)

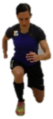

6–8 repetitions per leg, 2–4 sets.
Choose one of the following exercises (a, b, c):
  a. In-place lunges or Split Squat
  b. Walking lunges forward-backward
  c. Side lunges

6. Jumps (Speed & Deceleration)

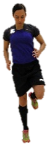

10–20 repetitions per leg, 2–4 sets.
Choose one of the following exercises (a, b, c):
  a. Single leg jumps over a line
  b. Skate jumps
  c. 3D jumps

7. Speed Run (Speed & Deceleration)

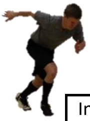

15–20 m, 2–4 laps.
Forward run progressing speed with controlled deceleration.
Walk back to the starting point.

In total, 40 exercise variations
Check out the videos of the NMT Warm-Up exercises here:
https://www.youtube.com/channel/UClm5f0iA3DZFjTWzdNs0ZLA
