# Supplementary material for: Adherence to an Injury Prevention Warm-Up Program in Children’s Soccer—A Secondary Analysis of a Randomized Controlled Trial
Source: Int J Environ Res Public Health. 2021 Dec 13;18(24):13134. doi: 10.3390/ijerph182413134 (PMC8701740; doi:10.3390/ijerph182413134)
Supplement: Supplementary file 1 [file ijerph-18-13134-s001.zip › Table S1.pdf]

Table S1. Number of weekly neuromuscular training warm-up sessions through follow-up.

Adherence group	Team	Week 1	2	3	4	5	6	7	8	9	10	11	12	13	14	15	16	17	18	19	20
High	Club C U14	4	4	4	4	3	4	3	2	4	1	2	2	2	3	2	3	3	3	1	3
High	Club D U12	2	3	2	3	3	2	1	3	3	3	2	3	3	4	2	3	3	3	4	3
High	Club D U14	5	5	1	1	5	2	4	3	3	4	2	2	2	1	1	0	1	2	2	0
High	Club H U14	2	2	3	2	3	3	2	2	2	2	2	2	2	2	2	3	2	3	2	2
High	Club D U11	1	2	2	3	4	2	0	3	2	2	2	2	3	3	1	2	3	2	3	0
High	Club I U14 (f)	4	3	3	4	1	3	2	2	2	2	1	2	1	1	3	2	2	2	2	0
High	Club B U13 (f)	3	2	3	2	0	2	3	2	3	3	2	2	2	2	2	2	4	1	0	0
High	Club H U12	2	3	2	2	0	2	2	2	1	2	2	3	3	0	1	3	3	3	1	3
High	Club E U11	2	3	2	2	2	0	2	2	3	2	2	1	2	2	2	2	2	2	2	2
High	Club E U13	2	2	2	2	2	0	2	2	2	2	2	2	2	2	2	2	2	2	2	2
High	Club G U13	3	3	2	3	0	3	2	3	3	2	1	0	2	2	1	2	2	1	2	1
High	Club A U11	2	2	2	2	0	1	2	2	2	2	2	2	2	2	1	2	2	3	2	2
High	Club I U12 (f)	2	2	2	2	0	2	2	3	2	2	2	2	2	2	1	2	2	1	2	2
High	Club B U13	0	1	1	2	2	2	2	2	2	2	2	2	2	2	2	2	2	2	2	2
High	Club B U11	0	2	2	2	1	2	2	2	2	2	1	2	2	2	2	2	2	2	2	2
High	Club B U11 (f)	3	3	3	3	0	2	3	3	2	2	2	2	2	0	0	2	1	1	1	1
High	Club E U14	0	0	3	2	3	0	2	3	3	2	1	2	2	2	1	1	1	3	2	3
Med	Club A U12	2	2	2	2	1	2	2	1	2	2	1	2	1	2	1	2	2	2	2	2
Med	Club I U11 (f)	2	3	3	2	1	0	2	2	1	2	1	2	2	1	1	2	2	1	3	2
Med	Club A U14	3	4	3	1	1	1	2	2	0	2	2	0	2	2	2	2	2	1	1	1
Med	Club F U14 (f)	2	2	2	2	2	0	2	2	2	2	2	2	2	1	1	2	2	1	2	1
Med	Club J U11	2	2	1	1	3	1	2	3	1	0	2	2	3	2	0	3	1	1	2	2
Med	Club B U12 (f)	0	2	3	3	1	0	2	2	2	2	0	2	1	2	3	2	2	2	2	0
Med	Club H U13	1	2	2	2	0	3	2	1	2	2	1	2	2	2	2	2	1	1	1	2
Med	Club F U12 (f)	2	2	2	2	2	0	2	2	2	2	1	1	2	1	2	2	1	1	1	2
Med	Club B U12	2	2	2	2	0	2	2	2	2	2	1	2	0	2	1	2	1	2	1	0
Med	Club D U13	5	4	1	1	3	1	1	0	1	2	1	1	0	0	1	2	2	2	1	1
Med	Club E U12	0	2	0	3	2	0	1	4	1	2	3	2	2	2	0	1	2	1	2	0
Med	Club I U13 (f)	2	2	3	3	0	2	2	3	1	2	1	2	1	1	1	1	1	1	1	0
Med	Club C U13	0	0	0	0	0	2	3	2	2	1	2	2	1	2	2	2	2	2	2	2
Med	Club A U13	2	2	2	2	2	2	2	2	2	0	0	0	0	2	1	2	1	3	1	0
Med	Club B U14 (f)	2	2	2	1	1	2	2	2	1	1	2	3	3	1	2	0	0	0	0	0
Med	Club F U13 (f)	0	1	1	2	1	1	2	2	3	2	2	1	2	2	3	0	0	2	1	0
Med	Club G U14	2	4	2	3	0	1	2	1	1	0	2	1	2	1	1	2	2	1	0	0
Med	Club G U12	2	2	2	0	0	2	2	1	2	2	0	2	1	1	1	1	1	2	2	2
Med	Club J U12	2	2	1	1	1	1	1	2	2	1	1	1	2	2	0	3	1	1	2	1
Med	Club C U11	2	1	2	2	0	2	2	2	2	1	0	1	2	2	0	1	2	1	1	1
Med	Club G U11	2	2	2	0	0	2	2	0	2	2	1	2	1	2	1	1	2	1	1	1
Med	Club H U11	2	1	1	1	0	1	1	1	1	0	1	2	2	2	1	2	2	3	1	2
Med	Club C U12	2	1	0	2	0	2	2	2	1	2	1	1	1	1	1	2	1	0	0	3
Low	Club B U14	1	2	2	2	0	1	2	2	2	1	1	1	1	2	2	1	0	1	0	0
Low	Club F U11 (f)	1	2	2	2	2	1	2	0	1	1	2	0	1	1	1	2	0	1	1	0
Low	Club J U14*	2	2	2	2	2	0	2	1	0	0	0	0	0	0	0	0	0	0	0	0
Low	Club J U13*	2	2	0	0	0	0	0	0	0	0	0	0	0	0	0	0	0	0	0	0

Club names (n=10) are pseudonymized into letters A-J (f) = females' team; *Reported use of physiotherapist-supervised injury prevention program outside study protocol.
